# Efficacy of sotrovimab on omicron BA.2, BA.4 and BA.5 subvariants of sars-cov-2 vs. other early therapies: a systematic review and meta-analysis of literature data

**DOI:** 10.3389/fimmu.2024.1295029

**Published:** 2024-01-30

**Authors:** Antonio Russo, Pierantonio Grimaldi, Mariantonietta Pisaturo, Lorenzo Onorato, Nicola Coppola

**Affiliations:** Department of Mental Health and Public Medicine - Infectious Disease Unit, University of Campania Luigi Vanvitelli, Naples, Italy

**Keywords:** COVID-19, monoclonal antibodies, early treatment, mortality, hospitalization

## Abstract

**Background:**

The aim of this meta-analysis was to ascertain whether sotrovimab was effective in reducing COVID-19 related hospitalization and mortality also in Omicron BA.2, BA.4 and BA.5 subvariants compared to other antivirals effective in index period.

**Methods:**

A systematic review and meta-analysis of Randomized Controlled Trials (RCTs) and observational studies comparing the efficacy of early treatment with sotrovimab compared to other early treatment effective in index period, antivirals or monoclonal antibodies (mAbs), in patients with COVID-19 during BA.2, BA.4, BA.5 waves, conducted in accordance with PRISMA guidelines. We searched MEDLINE, Google Scholar and the Cochrane Library. Mortality and hospitalization were defined as outcomes.

**Results:**

Four studies were included, allowing a meta-analysis of 8,041 patients. Meta-analysis showed no statistical difference between groups in hospitalization and mortality. Precisely, the RR of mortality showed no difference in the sotrovimab group compared to treatment with other drugs (OR 0.38, 95% CI 0.10-1.49, p<0.166). As regards the rate of hospitalization, no significant difference resulted between the patients treated with sotrovimab and those with other drugs (OR 1.66, 95% CI 0.41-6.66, p=0.477).

**Interpretation:**

In conclusion, this meta-analysis showed no significant difference between sotrovimab or other antivirals in reducing COVID-19 evolution in patients with a high risk of progression, considering both hospitalization and mortality.

## Introduction

In November 2021 a new Variant of Concern (VoC) of the severe acute respiratory syndrome coronavirus-2 (SARS-CoV)- 2, named Omicron (BA.1), appeared in South Africa. Soon afterwards, it spread globally substituting the previously dominating VoC Delta, causing an unprecedented rise in the number of cases. In March 2022, a new subvariant, named BA.2, started spreading at an even faster rate, gaining the role of the world’s dominating subvariant (1, 2). A substitution at the L452 residue of the BA.2 spike protein gave rise to BA.4 and BA.5 subvariants, declared in May 2022 as subvariants of interest by the World Health Organization (WHO) (3). In a pandemic phase in which the asymptomatic infections burden widely exceeds the severe COVD-19 presentations, it is crucial to soon identify which patients should undergo the COVID-19 specialist attention; the use of comorbidities is widely suggested to stratify patient’s progression risk, and old biomarkers such as C-reactive protein, d-dimer, ferritin, interleukin-6 and neutrophil/lymphocyte ratio are widely used by clinicians to establish an a-priori risk, moreover, new promising biomarkers, such as Interferon-inducible protein 10, Growth Arrest-Specific Gene 6, Osteopontin, Calcitonin Gene-related Peptide and SARS-CoV 2 RNA quantitative polymerase chain reaction on blood sample, are gaining evidences in favour of their use in a preliminary evaluation (4).

Sotrovimab is a human, recombinant monoclonal antibody able to bind SARS-CoV-2 spike protein. It was approved by the U.S. Food and Drug Administration (FDA) in May 2021 for emergency use in mild and moderate CoronaVIrus Disease-2019 (COVID-19) patients at risk of disease progression (5). Unlike most monoclonal antibodies (mAbs) used in the pre-omicron era, sotrovimab showed *in-vitro* efficacy on BA.1 (6). Since *in vitro* sotrovimab failed to demonstrate neutralizing power against BA.2 subvariant, the FDA withdrew their approval in April 2022 (7), while its use was to a variable extent kept across Europe, up to present days. However, recent data suggested that sotrovimab function does not uniquely rely on direct antiviral action as it is potentially able to interact with fragment crystallizable (Fc) gamma receptor and complement proteins; thus, it may determine an immune-system activation even when no spike protein binding is warranted, such as in BA.2, BA.4 and BA.5 subvariants enabling clinical efficacy (6).

The aim of the present meta-analysis was to ascertain whether sotrovimab was effective in reducing COVID-19 related hospitalization and mortality also in Omicron BA.2, BA.4 and BA.5 subvariant compared to other antivirals effective in index period.

## Methods

### Search strategy and selection criteria

A systematic review and meta-analysis of randomized controlled trials (RCTs) and observational studies comparing the efficacy of early treatment with sotrovimab compared to other early treatments, antivirals or mAbs in patients with COVID-19 during BA.2, BA.4, BA.5 waves. The study was conducted in accordance with PRISMA guidelines (8).

Two researchers (AR and PG) screened original reports using MEDLINE, Google Scholar and the Cochrane Library from January 1, 2022 up to April 30, 2023, involving both medical subject heading (MeSH) terminology and relevant keywords to identify articles that evaluate the efficacy of sotrovimab in patients with Omicron subvariant BA.2, BA.4 or BA.5. We chose this starting date considering that in March 2022 BA.2 became the world’s dominating subvariant (1, 2).

The following items were used to search the studies: “COVID”, “sotrovimab”. In addition, the reference lists of all studies retrieved as full papers were manually searched to identify any other study that might be eligible for inclusion.

All studies included had to fulfil the following characteristics and inclusion criteria: (a) to show original data from RCTs or observational studies; (b) to investigate the efficacy of sotrovimab versus other antivirals, mAbs in the early phase of SARS-CoV-2 infection by BA.2, BA.4, BA.5 variants or in the historical period of dominance of BA.2, BA.4, BA.5 variants in the specific geographical area; (c) to report at least one of the outcomes clearly defined: hospitalization, death up to 28 days after the start of the infection; (d) to be published in the English language as a full paper.

The historical period of dominance of BA.2, BA.4, BA.5 variants was defined as the periods in which, according to the national or international reports, these variants cumulatively exceeded 50% of SARS-COV-2 infections in the geographical area of the patients enrolled in the paper.

The exclusion criteria of the meta-analysis were: (a) meta-analyses, letters, reviews, meeting abstracts, or editorial comments; (b) duplicate publications or studies reporting duplicate data.

The authors of studies not reporting separate data for patients who received treatment with sotrovimab or other antivirals/mAbs in the period identified with a cumulative prevalence of BA.2, BA.4 and BA.5 more than or equal to 50% were contacted to retrieve the information.

Two researchers (AR, PG) independently screened all citations on the basis of the title, abstract and key words in order to identify potentially eligible articles. Reasons for the exclusion of any study were recorded independently. Thereafter, studies selected during the first screening were retrieved as full texts to be assessed for inclusion. In the case of disagreement, the reviewers re-evaluated the article together; if a consensus was not reached, a third author (NC) was consulted.

### Data analysis

Two authors (MP, AR) working independently extracted the data using a data-collection form previously established. The following relevant information was collected from every article included in the analysis: last name of the first author, year of publication, country where the population was enrolled, calendar period of enrolment, study design, sample size, baseline patient characteristics and occurrence of the endpoint evaluated in each treatment group. The corresponding author was contacted if additional data were needed to identify patients enrolled in the study. If more than one study enrolled the same patient population, only the most complete article was included in the analysis.

Two reviewers (PG and LO) independently performed the quality appraisal of each study. Risk of bias assessment of RCTs was conducted using the Cochrane Risk of Bias Tool (9). The Newcastle-Ottawa Scale (NOS) was used to assess the quality of observational studies (10). The articles based on the Newcastle–Ottawa Scale score were divided into three groups: 0–3 (fair), 4–6 (moderate), and 7–9 (good). In the case of discrepancies between the researchers the quality assessment was jointly re-evaluated. If a consensus was not reached, a third reviewer (NC) decided.

Mortality and hospitalization up to 28 days after the evidence of SARS-CoV-2 infection were the outcomes of this meta-analysis. Risk ratios (RRs) were used as the meta-analytic measure of association between therapy and the incidence of events. For each study, a proportion of patients with an event for the two therapeutic approaches were used to calculate RR using a 2x2 table.

Heterogeneity between the studies was assessed using the Q statistic and *I²*. *I*^2^ values between 25% and 49% indicated low heterogeneity, between 50% and 75% indicated moderate heterogeneity and a *I*^2^ value of 75% or above indicated high heterogeneity; a P value of Q statistic less than 0.10 was considered significant (11). Considering the different population size of the studies we chose to perform only random-effect size. If both-armed zero-event (BA0E) was present we included it when treatment effects were unlikely, but excluded it when there was a decisive treatment effect (12). In the latter case, a sensitivity analysis including BA0E was performed.

Where not specified, tests were two-sided, and P values <0·05 were considered significant. All statistical analyses were performed using Stata/IC, version 16 software (Stata Corporation, College Station, TX, USA) (13).

## Results

**Figure 1:**
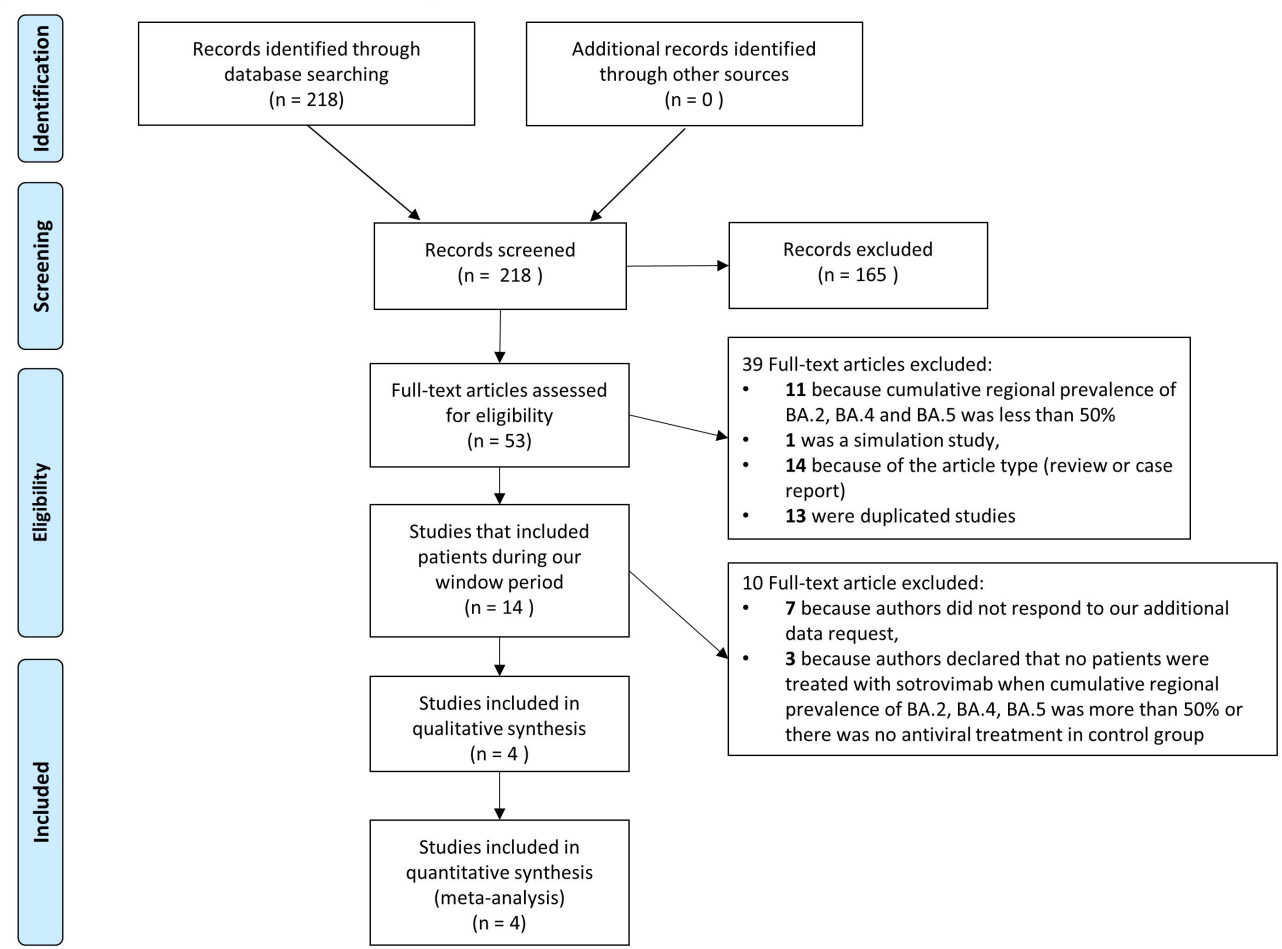
Flow chart of studies included. PRISMA flow diagram of the process of identification and selection of articles included in the meta-analysis.

The article identification and draft is shown in **Figure 1**. The authors identified a total of 218 citations from electronic search; among them, 165 were excluded on the basis of the title and abstract, and 39 for several causes (**Figure 1**). Fourteen papers included patients diagnosed with SARS-CoV-2 infection in the period in which, according to official data, a cumulative prevalence of BA.2, BA.4 and BA.5 variants greater than 50% occurred and were contacted for additional data: of these 14, 7 studies were excluded because authors did not respond to our request for data and 3 because the authors declared that no patients were treated with sotrovimab or there was no antiviral treatment in the control group. Finally, four studies were enrolled in the present paper: two studies that had been performed in Italy in a period with a high prevalence of BA.2, BA.4 and BA.5 variants according to the data of the Istituto Superiore di Sanità (ISS) (14) and for whom the authors answered our additional request properly (15, 16); one study that reported data on patients with only BA.2, BA.4 or BA.5 infection in a full article (17) and one study declared in the text to include patients with BA.2 and answered our additional request properly (18).

The characteristics of the 4 studies included are described in detail in **Table 1**. All studies were observational; two retrospective (15, 16), two prospective (17, 18). The patients enrolled in the studies ranged from 92 to 7,949, with a total of 6,130 patients treated with sotrovimab and 2,428 with other drugs. Precisely, of the 2,428 patients treated with other treatments, 2,150 were treated with molnupinavir, 135 with nirmatrelvir/ritonavir, 109 with remdesivir, 34 with casirivimab/imdevimab; no patient enrolled was not treated (**Table 1**)

**Table 1:** Characteristics of studies included in the meta-analysis considering the period when BA.2, BA.4 and BA.5 cumulative prevalence was more than 50%.

First Sauthor	Year	Country	Study design	BA 2-4-5 >50% prevalence following regional data	Control	N° of patients in sotrovimab	N° of patients in control group
De Vito et al. (14)	2023	Italy	Retrospective	Since April 9, 2022	Antivirals, Other Mabs	71	105 (molnupiravir), 28 (nirmatrelvir/ritonavir), 71 (remdesivir), 17 (casirivimab/imdevimab)
Scaglione et al. (15)	2022	Italy	Retrospective	Since February 5, 2022	Antivirals, Other Mabs	37	17 (casirivimab/imdevimab), 38 (remdesivir), 75 (molnupiravir), 58 (nirmatrelvir/ritonavir)
Zheng et al. (16)	2022	UK	Perspective Cohort	The study had a dedicated subanalysis.	Molnupiravir	5,979	1970 (molnupiravir)
Martin-Blondel et al. (17)	2022	France	Perspective Cohort	The study declare to include BA.2 patients	Nirmatrlevir/ritonavir	43	49(nirmatrelvir/ritonavir)

Quality assessment performed using the Newcastle-Ottawa Scale were reported in **Supplementary Material Table 1**. Two studies showed good quality (17, 18), two studies showed moderate quality (15, 16).

**Figure 2:**
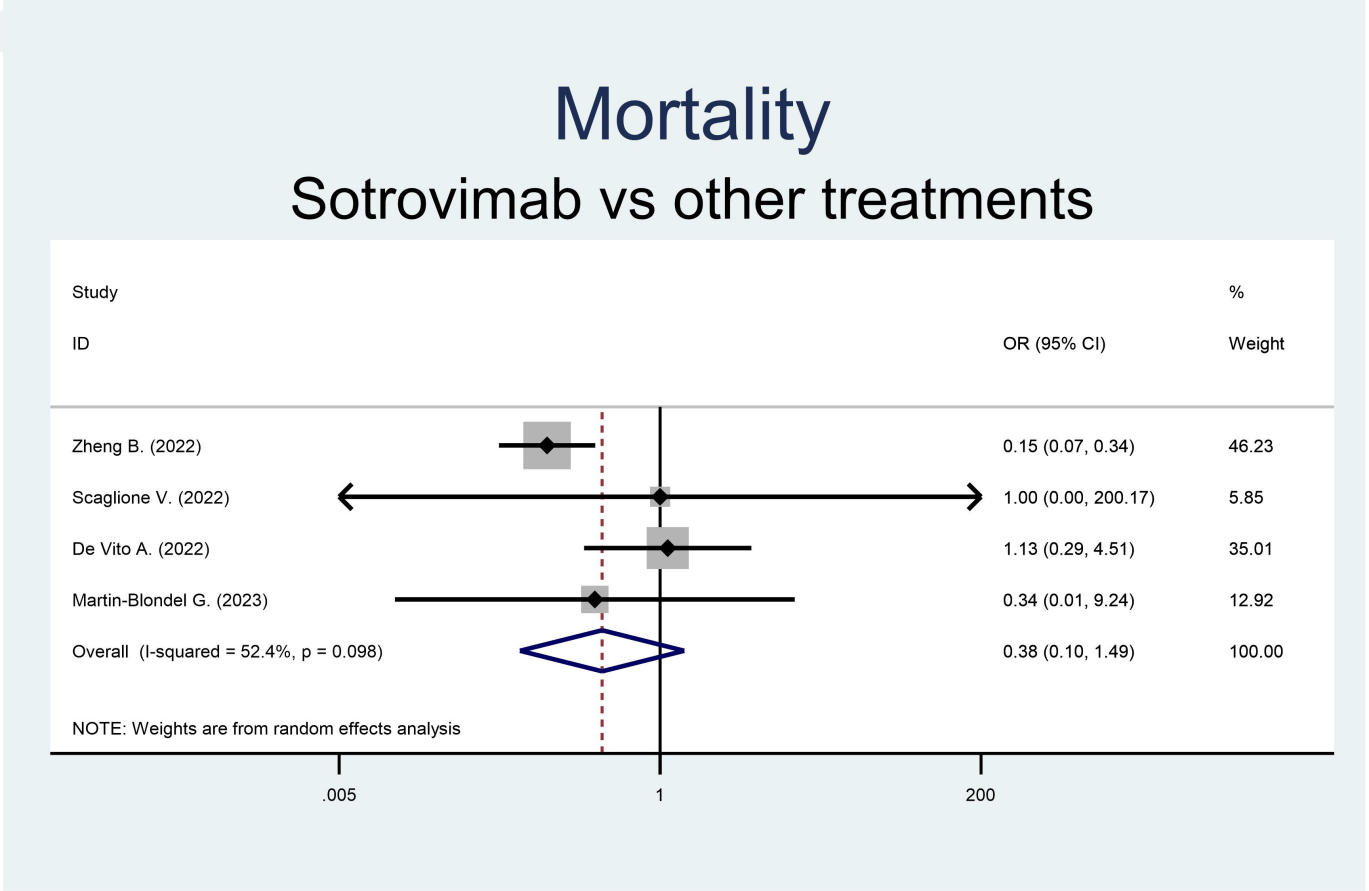
Forest plot of RRs of mortality in patients receiving sotrovimab or other antivirals.

**Figure 3:**
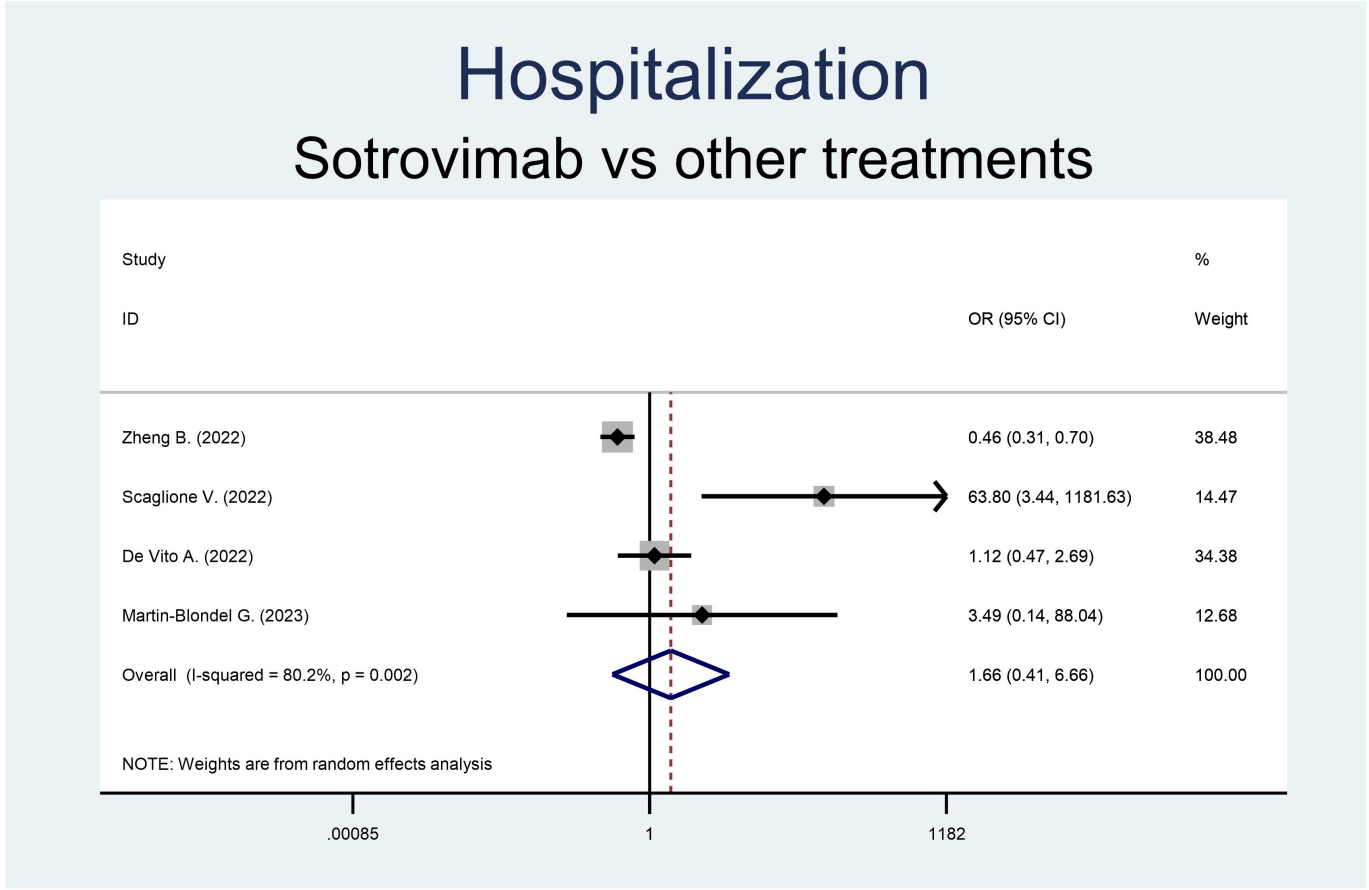
Forest plot of RRs of hospitalization in patients receiving sotrovimab or other antivirals.

Both the rate of hospitalization and mortality were similar in the groups (**Figures 2**, **3**). Precisely, the RR of mortality showed no difference in the sotrovimab group compared to treatment with other drugs (OR 0.38, 95% CI 0.10-1.49, p<0.166; **Figure 2**). As regards the rate of hospitalization, no significant difference resulted between the patients treated with sotrovimab and those with other drugs (OR 1.66, 95% CI 0.41-6.66, p=0.477, **Figure 3**). High heterogeneity was observed analyzing hospitalizations (*I*^2 ^= 80.2%, p=0.002), while a moderate heterogeneity was observed analyzing mortality (*I*^2 ^= 52.4%, p=0.098).

## Discussion

Monoclonal antibodies (mAbs) targeting the spike protein were largely used in patients with mild-moderate COVID-19 infection, improving clinical outcome and reducing mortality (19–24), representing a cornerstone for the early treatment of COVID-19 in patients with a risk of severe illness.

Recently, bebtelovimab and sotrovimab were not authorized for emergency use in the United States considering the reduced activity against Omicron variants, in particular against BQ.1 and BQ.1.1 for bebtelovimab and BA.2 for sotrovimab. To date mAbs are not authorized by the National Institute of Health (NIH) (25–27) but are currently allowed by the European Medicine Agency (EMA) (28). In fact, while in the early Omicron era, sotrovimab showed efficacy compared to other antivirals (remdesivir, nirmatrelvir/ritonavir, molnupiravir) (15–18), while *in vitro* and clinical studies showed reduced activity of sotrovimab against Omicron BA.2 (29).

In the present meta-analysis we aimed at investigating the efficacy of sotrovimab in reducing COVID-19-related hospitalization and death in a period when the prevalence of Omicron variants BA.2, BA.4, BA.5 were more than 50%, compared to others drugs for early treatment considered effective for variants and appropriate for patients’ clinical conditions. In our study, patients treated with sotrovimab showed a similar rate of COVID-19-related hospitalization and mortality.

It is not easy to explain the disagreement between the *in-vitro* and clinical data on the efficacy of sotrovimab on BA.2, BA.4, BA.5 variants. However, in July 2022 the study of Case JB et al. showed a different mechanism of protection against BA.2 by sotrovimab, which utilizes the Fc effector function interaction rather than direct neutralization (30). The activity mediated by the Fc effector function could be the key to understanding its efficacy even in the cases of reduced or absent *in vitro* neutralizing activity of sotrovimab.

Despite the worldwide reduction in the number of cases of SARS-CoV-2 infection, early therapies are still, nowadays, a necessary treatment to reduce the clinical progression of COVID-19. Considering the settings of patients at a high risk of progression and comorbidities and the pharmacological treatment to which the patients are subjected, they may not be eligible for antivirals. Monoclonal antibodies, given the almost absence of drug interactions and the possibility of administering them to patients with severe hepatic and renal impairment, could represent a chance for these patients. In addition, highly immunocompromised patients may have longer viral shedding, reducing the chances of obtaining treatment for their underlying disease, often oncohematological, or that can improve the quality of life or survival. Mikulska M. et al. showed that in a cohort of patients with long viral shedding, those who were administered combination treatment including two antivirals and one mAb were more likely to have a higher rate of virological and clinical response compared to those who were administered only antivirals (31).

Our study has several strengths; firstly, a relatively large sample of patients were included in the analysis (6,130 patients treated with sotrovimab and 2,428 patients treated with other antivirals or mAbs). Furthermore, the outcomes analyzed, mortality and hospitalization, were clinically relevant and were reported in all the studies included.

However, there are some limitations. Considering our request for additional data, only 6 authors responded to our request. Treatment performed was various, but all treatments were considered in line with the recommendations issued by the respective supervisory organizations. The majority of patients included derived from a single study comparing sotrovimab to molnupiravir counting more than 1,970 patients in control group. This could lead to generalizability issues, but it is still necessary to consider that at the time of treatment molnupiravir such as nirmatrelvir/ritonavir or remdesivir were considered effective for early treatment. In addition, the limitations related to the type of study carried out were: lack of granularity, residual cofounding, heterogeneous study designs and heterogenous populations.

In conclusion, the present meta-analysis showed no significant difference between sotrovimab and other antivirals considered effective for variants and appropriate for patients’ clinical conditions in reducing COVID-19 evolution in patients with a high risk, considering both hospitalization and mortality

## Data availability statement

The raw data supporting the conclusions of this article will be made available by the authors, without undue reservation.

## Author contributions

AR: Data curation, Formal analysis, Investigation, Methodology, Writing – original draft. PG: Data curation, Investigation, Methodology, Writing – original draft. MP: Investigation, Supervision, Writing – review & editing. LO: Methodology, Supervision, Writing – review & editing. NC: Conceptualization, Supervision, Writing – review & editing.
